# From abstracts to published articles: Assessing the scientific publications from the annual meetings of the German Spine Society (DWG) in 2017, 2018, 2019, and 2022

**DOI:** 10.1016/j.bas.2025.104242

**Published:** 2025-04-25

**Authors:** T. Pantel, Jihoon On, S. Hackel, V. Hubertus, S. Ille, M. Kalbitz, J. Keller, J.H. Klingler, J. Onken, H. Schmidt, A. Younsi, S. Zwingenberger, M. Czabanka, P. Vajkoczy, M. Mohme, the Spine Science Commission of the German Spine Society (DWG)

**Affiliations:** a Department of Neurosurgery, University Medical Center Hamburg-Eppendorf, Hamburg, Germany; b Department of Orthopaedic, Inselspital Bern, Bern, Switzerland; c Department of Neurosurgery, Charité – Universitätsmedizin Berlin, Corporate Member of Freie Universität Berlin, Humboldt-Universität zu Berlin, Berlin Institute of Health, Germany; d Berlin Institute of Health (BIH) Charité Clinician Scientist Program, Germany; e Department of Neurosurgery, University Hospital Heidelberg, Heidelberg, Germany; f Department of Trauma and Orthopedic Surgery, University Hospital Erlangen, Erlangen, Germany; g Department of Trauma and Orthopedic Surgery, University Medical Center Hamburg-Eppendorf, Hamburg, Germany; h Department of Neurosurgery, University Hospital Freiburg, Freiburg, Germany; i Julius Wolff Institut, Berlin Institute of Health - Charité at Universitätsmedizin Berlin, Berlin, Germany; j University Center of Orthopedic, Trauma and Plastic Surgery, University Hospital Carl Gustav Carus at TU Dresden, Germany; k Department of Neurosurgery, University Hospital Frankfurt, Frankfurt, Germany

**Keywords:** Scientific congress, Spine, Research, Publication rate

## Abstract

**Introduction:**

The annual meeting of the German Spine Society (DWG) serves as a platform for discussing the latest academic findings. This study assessed the scientific quality of these conferences by investigating the publication rate of abstracts presented at DWG Annual Meetings (2017–2019, 2022) in peer-reviewed journals and analyzing factors influencing publication rates.

**Research question:**

How did the publication rates of the annual meeting of the DWG evolve, and what factors influenced them.

**Material & methods:**

We reviewed all accepted oral and poster presentations and conducted a PubMed database search (up to 10/2023) to determine publication rates, time to publication, research type, and topic distribution.

**Results:**

A total of 730 abstracts were presented, with 275 (37.6 %) published in peer-reviewed journals: 27.9 % (2017), 52.7 % (2018), 39.1 % (2019), and 29.9 % (2022). A subset of 61 (8.4 %) abstracts had already been published before the conference. The mean time to publication was 35.4 ± 22.3 months. Basic research and experimental studies constituted 22.1 % of abstracts, achieving a 44.1 % publication rate. Key research fields included spinal cord injury (16.9 %), biomechanics (14.8 %), spinal oncology (8.8 %), and bone and cartilage (4.4 %), while 55.1 % covered other spinal surgery topics.

**Discussion & conclusions:**

With 37.6 % of abstracts published in PubMed-indexed journals, our findings highlight the scholarly impact of DWG meetings. These results reflect the scientific quality of submissions and provide insights for future improvements to enhance the DWG's research focus.

## Highlights

• The DWG Annual Meeting aims to discuss the latest academic findings and assess the scientific quality of presented research.
• Of 730 abstracts presented at DWG meetings (2017–2019, 2022), 37.6 % were published in peer-reviewed journals.
• The publication rate varied across years, with the highest in 2018 (52.7 %) and the lowest in 2017 (27.9 %).
• Basic research and experimental studies accounted for 22.1 % of abstracts, achieving a publication rate of 44.1 %.
• The findings highlight the scholarly impact of the DWG and provide direction for future scientific development.

## 1 Introduction

Annual meetings of medical societies are a unique forum for the presentation and discussion of new scientific findings, intended to support the research approach and promote further development of the medical field (Whitehouse et al., 2009). The objective of the annual meeting of the German Spine Society (DWG) is to unveil the latest insights into spinal surgery. To achieve the highest possible scientific quality of the research presented, papers were selected in a blinded review process prior to the conference. The members of this committee are long-standing academically distinguished members of the DWG. The abstracts accepted for the presentation were published in advance in the European Spine Journal.

The aim of the respective authors is usually to create a high-quality scientific publication from a congress presentation, which is then published in a peer-reviewed journal. This process broadens the accessibility of scientific findings to a more extensive audience than conference presentations alone. The successful development of a scientific publication is ultimately not only important for the author but also reflects the quality of the respective conference (Whitehouse et al., 2009). As the respective medical societies become increasingly aware of this, several studies on this topic have been carried out and published by individual societies in recent years (Czorlich et al., 2016; Halickman et al., 2018; Janssen et al., 2016; Patel et al., 2011; Schulte et al., 2012). The reported publication rates vary considerably in some cases, which may be due to various reasons (Janssen et al., 2016; Schulte et al., 2012).

The German Spine Society (DWG) has set the goal of steadily increasing the scientific profile of its annual conference in the coming years and establishing itself as an internationally visible scientific platform for spine surgery. With the founding of the Spine Science Commission, the society is breaking new ground and creating a firmly anchored forum within the society that is dedicated to scientific development. Against this background, this study aims to investigate how successfully congress papers from the annual meeting of the German Spine Society (DWG) are published in peer-reviewed journals. We also want to examine whether specific research fields, study design, or type of presentation increase the likelihood of publication, as described in other papers.

## 2 Methods

The abstracts of the 2017–2019 and 2022 annual meetings published in the European Spine Journal were retrospectively analyzed. For each abstract, various items necessary for scientific classification were recorded (Table 1). A PubMed search was performed for each abstract (end date: 10/2023). The search was conducted using the names of the first or last author and at least two scientific keywords from the abstract title. The abstracts were assessed as published if the data matched between the two abstracts. If no clear assignment was possible (e.g., disagreement between authors, different main focus between abstract and paper), the abstract was classified as unpublished. The Journal Impact Factor (JIF) were determined based on the year of publication (https://jcr.clarivate.com). The publication rate was determined for all evaluated conferences, as well as for the respective conferences. In addition, subgroup analyses were performed for presentation type (poster/lecture), study type (prospective/retrospective), and the research area of the article (oncology, cartilage/bone, biomechanics, spinal cord injury, and clinical study) (see Table 2).

**Table 1:** Detailed overview of the number of scientific contributions per congress year and corresponding subgroup.

	2017	2018	2019	2022	total
**Presented abstracts**					
Overall	165	182	199	184	730
Poster	107	109	128	105	449
Talk	58	73	71	79	281
Best of	10	7	9	8	34
**Study design**					
Retrospective	108	100	123	94	425
Prospective	25	44	34	43	146
Others^a^	32	38	42	47	159
**Research type**					
Basic science	52	29	37	43	161
clincical	113	153	162	141	569
**Research field**					
Spinal oncology	18	13	19	14	64
Cartilage/Bone	8	9	9	6	32
Biomechanics	27	29	30	22	108
Spinal cord injury	26	35	27	36	124
others	86	96	114	106	402
**Submitting discipline**					
Orthopaedics/trauma surgery	86	99	121	112	418
Neurosurgery	42	71	72	68	253
others	37	12	6	4	59

a Reviews, Guideline, Survey.

**Table 2:** Detailed overview of the proportion of successfully published articles per congress year and corresponding subgroup.

	2017	2018	2019	2022	total
**Presented abstracts**					
Overall	46/165 (27.9)	96/182 (52.7)	78/199 (39.1)	55/184 (29.9)	275/730 (37.6)
Poster	22/107	50/109	45/128	28/105	104/449
Talk	24/58	46/73	33/71	27/79	171/281
Best of	2/10	2/7	2/9	2/8	8/34
**Study design**					
Retrospective	26/108	41/100	35/123	26/94	128/425
Prospective	8/25	27/44	22/34	14/43	71/146
Others ∗	12/32	28/38	21/42	15/47	76/159
**Research type**					
Basic science	18/52	20/29	21/37	12/43	71/161
clinical	37/113	58/153	75/162	34/141	204/569
**Research field**					
Spinal oncology	4/18	4/13	8/19	7/14	23/64
Cartilage/Bone	4/8	5/9	5/9	1/6	15/32
Biomechanics	9/27	18/29	12/30	5/22	34/108
Spinal cord injury	9/26	18/35	11/27	14/36	52/124
others	86	96	114	106	402
**Submitting discipline**					
Orthopaedics/trauma surgery	30/86	52/99	48/121	28/112	158/418
Neurosurgery	8/42	34/71	27/72	27/68	96/253
others	8/37	10/12	3/6	0/4	21/59

### 2.1 Statistical analysis

GraphPad Prism 9 was used for statistical analyses and graphs. The publication rates of the different years were compared using the chi-square test. Statistical significance was set at P ≤ 0.05.

## 3 Results

A total of 730 scientific presentations were given at the DWG Annual Meetings of 2017–2019 and 2022. Of these contributions, 449 (61.5 %) were presented in the form of poster presentations, while the remaining 281 (38.5 %) were presented as lectures. 34 (4.7 %) of the total contributions were presented as part of the "best-of-session" as they were rated as outstanding contributions. The study design of the contributions presented was mostly clinical retrospective (n = 425; 58.4 %). Broken down by subject area, most of the contributions (n = 124; 16.9 %) dealt with the area of spinal cord injury (SCI), followed by spinal biomechanics, which was the subject of 108 (14.8 %) of the contributions. Spinal oncology was covered in 64 (8.8 %) and cartilage/bone in 32 (4.4 %) of contributions. The other 402 contributions (55.1 %) dealt with various topics such as degenerative pathologies or deformities (55.1 %), respectively. In addition, 161 (19.5 %) contributions to basic science were presented. A total of 418 (57.3 %) contributions were submitted by trauma surgeons or orthopedic or trauma surgeons, and 253 (34.7 %) were submitted by neurosurgeons. The remaining contributions have been submitted to various disciplines. Table 1 provides a detailed overview of this study.

**Fig. 1:**
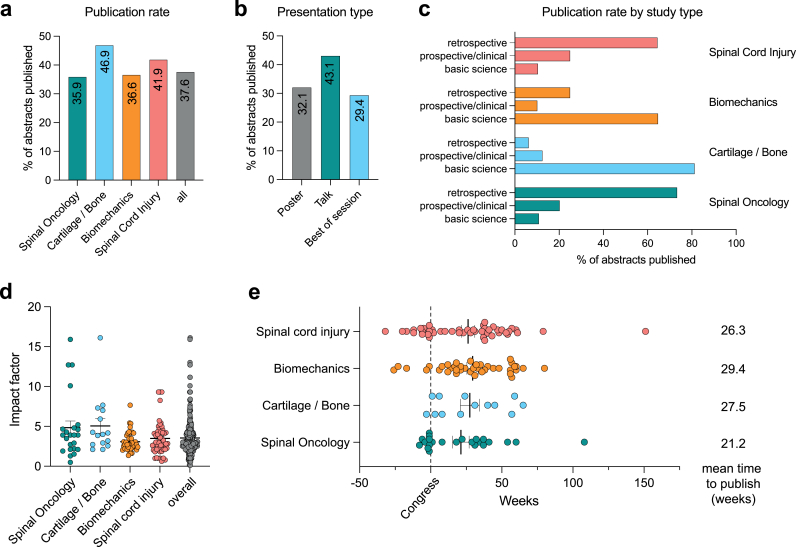
Detailed overview of publications rates, impact factor distributions and publication duration.

Overall, 275 (37.6 %) of all contributions were published in peer-reviewed journals (27.9 % (2017), 52.7 % (2018), 39.1 % (2019) and 29.9 % (2022)). Oral presentations were published more frequently than poster contributions (43.1 % vs. 32.1 %; p < 0.05). The Journal Impact Factor (JIF) for all published contributions was 3.54 ± 2.33. Successfully published articles in the field of spinal oncology achieved a JIF of 4.84 + 4.05. Biomechanical articles were published in peer-reviewed journals with a JIF of 5.06 + 3.08. Papers on cartilage/bone and spinal cord injuries achieved a JIF of 3.08 ± 1.21 and 3.49 ± 2.07, respectively (Fig. 1).

The time from congress presentation to successful publication in a peer-reviewed journal was 34.25 ± 22.30 months. There was no significant difference in the durations of the individual scientific subcategories. A total of 61 (8.4 %) articles were successfully published prior to the congress presentation (10.87 ± 17.34 months) (Fig. 1).

## 4 Discussion

Medical-scientific conferences aim to provide a presentation and discussion platform for new findings in the respective specialty and thus contribute to the further development of the specialist field (Whitehouse et al., 2009). The manner in which the quality of scientific congresses should be assessed remains a subject of debate (Schulte et al., 2012). One marker for assessing the scientific quality of a conference is the rate of successfully published papers in peer-reviewed journals that were initially presented at the conference (Czorlich et al., 2016; Scherer et al., 2007). The fact that the rating of scientific quality is of increasing relevance for the respective medical societies is reflected in the accumulation of comparable evaluations that have been published in recent years (Whitehouse et al., 2009; Czorlich et al., 2016; Halickman et al., 2018; Janssen et al., 2016; Patel et al., 2011; Schulte et al., 2012; Scherer et al., 2007; Harvey and Wandersee, 2010; Yilmaz et al., 2013; Wang et al., 1999).

Evaluation of the aforementioned DWG annual meetings revealed an overall publication rate of 37.6 %, which is comparable with published evaluations of other spine conferences (range: 37.8 %–54 %) (Schulte et al., 2012). Additionally, the publication rate of our analysis is comparable to that of an annual neurosurgical conference (German Neurosurgical Society, DGNC) without a specific focus on spinal surgery (37.6 % vs. 40.4 %) (Czorlich et al., 2016). Focusing on the special subgroup of articles from the "spine" section of this conference, we found an almost identical publication rate of 36 % (Czorlich et al., 2016). A systematic analysis of the publication probability of scientific conference proceedings by the Cochrane Collaboration in 2007 yielded a slightly higher publication rate (44.5 %) (Scherer et al., 2007). Another aspect frequently mentioned in the context of a successful publication is the format of the conference contribution (poster presentation or lecture). Accordingly, we have included this information in the analysis. Our data showed that oral presentations were published much more frequently than poster contributions (43.1 % vs. 32.1 %). Other available studies on this topic show a similar result, with only one reporting that poster contributions were successfully published more frequently than oral presentations. In our study, prospective studies and basic research performed better than retrospective or clinical studies in terms of the publication rate. This underlines the results of Schulte et al. (2013), who reported similar findings in their evaluation of the Annual Congress of the Spine Society of Europe (Schulte et al., 2012).

Several potential factors can influence publication rates. In our view, a key factor that determines scientific quality is the blinded review carried out in advance of each conference, in which various scientifically active spine surgeons are involved. The publication rates for the conferences and review processes mutually confirm their quality. In turn, the underlying type of presentation at a conference as an influencing factor for the likelihood of publication is controversial. In our analysis, lectures were published significantly more often in peer-reviewed journals than poster presentations. This is consistent with the findings of Schulte et al. and Preston et al. (Schulte et al., 2012; Preston et al., 2005). A common assumption as a potential explanation is that, for lectures, mostly abstracts that are rated as having higher scientific quality are accepted. Assuming that this is an accurate conclusion, the importance of the review process in the run-up to congress is underlined. However, the publication rate of 32.1 % of the poster contributions in our study is also good; therefore, the assumption of a lower scientific quality of these contributions is refuted in our eyes. In our analysis, the study design (in addition to the form of presentation) had a significant influence on publication rate. This is also consistent with data from other studies (Patel et al., 2011; Schulte et al., 2012; Yilmaz et al., 2013). In addition to the form of presentation, the study design also had a significant influence on the publication rate in our analysis, which is consistent with data from other studies.

The median time to successful publication following the congress presentation in our study was 34.25 ± 22.3 months, which tends to be somewhat longer compared to other analyses (Schulte et al., 2012). Ultimately, it must be kept in mind that the submitting colleagues are usually clinically active surgeons and conduct research alongside their clinical work, which certainly affects the speed of publication. The extent to which the review and publication processes of the respective journals contribute to the length of time can certainly be controversial.

The mean Journal Impact Factor (JIF) of all published abstracts in our analysis was 3.56 ± 2.3. No significant difference was found between the various subgroups (lecture, poster, prospective/retrospective study, etc.), but rather, a very homogeneous JIF distribution. Therefore, a higher publication rate in individual subgroups is not necessarily associated with a higher JIF. It is striking that the JIF was significantly better than that reported in other studies. Czorlich et al. report in their study a mean JIF of 2.99 ± 3.3, and specifically for the spine section the JIF was 1.89 (Czorlich et al., 2016). This was significantly lower than that observed in the present study. The same applies to data from Schulte et al., who reported a mean JIF of 1.79 (Schulte et al., 2012). One reason for the significantly higher mean JIF in our study could be the increasing relevance of spinal surgery issues and the associated increase in citation frequency in recent years. Another possible explanation for the higher JIF could be that the JIF of journals has increased considerably in recent years, which limits comparability with older studies (Baethge, 2024).

Like every study, ours also has limitations that need to be considered when interpreting the results and that we would therefore like to address. First, our study is potentially limited by the scope of the PubMed database used for our search (Nourbakhsh et al., 2012). An additional search in other databases, some of which have an even larger scope, could have led to a higher number of search results and, thus, a higher publication rate. Another possible limiting factor is the growing number of new scientific journals that are currently being established but are not yet listed in PubMed. Ultimately, however, all studies on this topic must deal with this problem; therefore, the comparability of the data between the studies is not limited, owing to the identical search pattern.

Despite the high scientific quality of the conference, measured by the final JIF of publications initially selected as abstracts, the publication rate per se remains low, just over a third. Although this is comparable to similar publications at other conferences, this fact should also be questioned. In principle, it can be assumed that scientific studies are submitted as abstracts, of which two-thirds are not published within the period covered. The present data do not allow for a fundamental analysis of the reasons for this.

In summary, we conclude that a publication rate of 37.6 % is a good result, which is in line with the values reported by other studies on this topic. The median JIF of 3.56 ± 2.3, on the other hand, was significantly higher than that in comparable studies. In our view, both reflect a good scientific quality of conference submissions, while also indicating the scholarly quality of the submissions and the meeting itself, as well as defining future goals to further develop the scientific focus of the DWG.

## Declaration of competing interest

The authors declare the following financial interests/personal relationships which may be considered as potential competing interests: Sebastian Ille reports a relationship with Brainlab AG, Icotec AG, Carl Zeiss Meditec AG, Nexstim AG that includes: consulting or advisory. 10.13039/100002376VH was funded by the 10.13039/501100017268BIH Charité Clinician Scientist Program (10.13039/501100017268Berlin Institute of Health). If there are other authors, they declare that they have no known competing financial interests or personal relationships that could have appeared to influence the work reported in this paper.
